# Analysing the behaviour change techniques in an effective food literacy program to inform future program design

**DOI:** 10.1111/1747-0080.12908

**Published:** 2024-10-22

**Authors:** Lucy M. Butcher, Caylah Batt, Sophie Royce, Eamon Barron, Roslyn Giglia, Andrea Begley

**Affiliations:** ^1^ Edith Cowan University Joondalup Western Australia Australia; ^2^ Foodbank Western Australia Perth Airport Western Australia Australia; ^3^ Curtin University Bentley Western Australia Australia

**Keywords:** behaviour change techniques, cooking, education, program evaluation

## Abstract

**Aim:**

Food literacy programs aim to improve food related skills and knowledge and are selected Governments as a strategy to address dietary intakes at a community level. The purpose of this research is to identify behaviour change techniques in a food literacy program, which were assessed by the achievement of participant goals related to food literacy and dietary behaviour changes.

**Methods:**

Consensus methods were applied to map behaviour change techniques to an adult food literacy program. A second phase investigation applied content analysis of participant process evaluation data (collected between 2016 and June 2021) to align target behaviours and behaviour change techniques. Chi‐square and ANCOVA were used to assess the statistically significant demographic characteristics, food literacy, and dietary behaviour scores for participants who set goals and recorded changes.

**Results:**

A total of 4697 program participants provided evaluation data from the 4‐week food literacy program. Participants who set goals and made changes were statistically more likely to have higher food literacy factor scores (*p* < 0.001) and fruit serves (*p* = 0.004). Statistical analysis showed that participants were more likely to have set goals and recorded changes if they were female (*p* < 0.001), older (*p* < 0.001), higher education level (*p* < 0.001), had a higher socioeconomic status (*p* = 0.049), lived with children (*p* = 0.014), were born in Australia (*p* = 0.019), or did not identify as Indigenous (*p* < 0.001). The behaviour change technique mapping process identified nine techniques used weekly and a total of 22 techniques used over the entire 4 week program curriculum.

**Conclusions:**

This is the first Australian study to link the contribution of behaviour change techniques to food literacy and dietary behaviour change in an established effective food literacy program. Knowing the behaviour change techniques associated with effective programs will facilitate replication of effective interventions.

## 1 INTRODUCTION

Investment in food literacy programs has increased as a strategy for improving dietary behaviour.^1^ These programs assist people in planning, selecting, preparing, and emphasising the cooking skills required to eat healthy foods, acknowledging food literacy as a determinant of dietary behaviour change. Training style or education based food literacy interventions have demonstrated positive outcomes regarding knowledge, improved consumption behaviours and heightened social connection^2^, ^3^, ^4^, ^5^, ^6^ Theoretically driven evidence‐based programs are more likely to change dietary behaviour.^7^ However, there is limited evidence that food literacy programs are designed with an underlying theoretical framework or model. Evidence reviews by McGowan, Caraher^8^ and Hollywood, Surgenor^6^ of food and cooking skill interventions, found that less than a third of the interventions identified outlined an explicit theory.^6^ A more recent review of culinary interventions in older adults found that 18 of 39 studies used behavioural theory.^9^ Even when theory use is acknowledged, details are lacking on how it has been appropriately applied to curriculum design, the development of learning strategies, and the initiation and maintenance of behaviour change.^1^, ^8^ The exact components that promote behavioural change within food literacy programs remain unclear.

Nutritionists and dietitians can apply behavioural science to explore what enables behaviour, what prevents, and how to influence behaviours impacting health outcomes in the context of everyday life. The primary objective of food literacy programs is to improve food literacy knowledge, skills, and use, which are indirectly measured; therefore, it is important to elicit methods about how a program changes these behaviours.^10^ Behaviour change techniques are small intrinsic components of an intervention that are observable, replicable, and have the potential to bring about change.^11^ The Behaviour Change Technique Taxonomy (v1) was developed by Michie et al. to provide standardised definitions for 93 behaviour change techniques clustered into 16 groups^12^ representing targets of capability, opportunity, and/or motivation to change behaviour. The Behaviour Change Technique Taxonomy provides a useful framework for identifying the behaviour change techniques present in interventions to classify the potential mechanisms or ‘active ingredients’ within the program content associated with effectiveness.^12^ In diet‐related interventions systematic reviews of behaviour change techniques there is evidence of intervention features that deliver various behaviour change techniques. For example, a scoping review of behaviour change techniques in digital health interventions designed to improve diet in low socioeconomic status groups found an average of 6.9 behaviour change techniques (range, 3–15) in 17 studies but no clear association with the number or type of behaviour change techniques used for the target group in effective studies.^13^ Overall, there is differential use, lack of or ambiguous recording of behaviour change techniques which limits comparison and development of dietary behaviour change techniques taxonomies.^14^ There have only been a few attempts to identify behaviour change techniques in food literacy programs, including a systematic review^6^ and a realist synthesis examining the context and mechanisms.^15^ However, mapping the Behaviour Change Technique Taxonomy to program content may be able to identify the active ingredients more precisely than systematic reviews reporting content in publications for determining behaviour change techniques. Goal setting, for example, has long been established as a technique to support dietary behaviour change, but evidence of its effectiveness in food literacy programs is limited.^16^ There are only two instances where behaviour change technique mapping exists for individual food literacy programs^17^, ^18^ and only one of these programs has been evaluated.^17^

Food literacy programs are education‐ and training‐type interventions in the Behaviour Change Wheel of interventions to select from to address target behaviours.^11^ Education programs typically use behaviour change techniques that inform (e.g., awareness of food groups), explain (e.g., consequences of not eating the five food groups), show (e.g., how to select healthier foods by reading food labels), and provide feedback (e.g., achievement of healthy eating goals) to increase knowledge and understanding. Hands‐on training, such as cooking, uses behaviour change techniques related to demonstration (e.g., safe hand washing), supervision (e.g., showing how to start a new recipe), and support practice with feedback (e.g., tasting a new recipe) to improve cognitive and physical skills. Education and training interventions can also build habits (e.g., repeated cooking and practice at home). Therefore, it is expected that an effective food literacy program over several weeks with hands‐on cooking would match several behaviour change techniques, while identifying and analysing behaviour change techniques employed in effective food literacy programs is important to enable the replication of successful future interventions.^19^

Understanding whether a program is effective is central to evaluation; however, it is also necessary to identify how and why a program works. Process evaluation informs how programs are implemented and provides insights into uptake, acceptability and use.^20^ Knowing what participants find useful and respond to is essential, as it identifies the successful and unsuccessful program components to improve program improvements. It can also identify the barriers and facilitators in the implementation process. Despite their importance, few food literacy programs have published process evaluations^1^, ^21^, ^22^ indicating that they are neither systematically performed nor reported alongside effectiveness studies.

It is critical to understand how food literacy programs supported by government investments actively bring about improvements in population health. This requires going beyond listing the theoretical perspective to uncover the behaviour change techniques that need to be used to produce positive changes in food literacy and, ultimately, dietary behaviours. Identifying combinations of behaviour change techniques has the potential to be effective, as robust evidence of food literacy program effectiveness is lacking.

The purpose of this research is to (1) identify the behaviour change techniques in a food literacy program lesson content, (2) explore goals participants' set at the start of the program, (3) assess which participants were likely to set goals and if goal setting was associated with food literacy and dietary behaviour changes, and (4) describe how the behaviour change techniques were evident in their process evaluation responses.

## 2 METHODS

The Food Sensations® for Adults program was a nutrition education and cooking program delivered in the Western Australian (WA) community. The program was funded by the WA Department of Health and was delivered by the Foodbank WA between 2016 and 2022. The program had a demonstrated capacity to promote sustained improvements in food literacy and dietary behaviours in adults from low‐to middle‐income households, the primary target audience.^23^, ^24^, ^25^

The program was designed using both the Health Belief Model and the Social Cognitive Theory. All Health Belief Model constructs were incorporated into the curriculum: perceived benefits and health motivation for action, and to identify and reduce perceived barriers to planning, selecting, preparing, and eating healthy foods by developing skills, including enhancing confidence in the ability to succeed (self‐efficacy). Additionally, the lesson content enabled participants to build on a perceived threat to disease by describing the consequences (perceived susceptibility) and beliefs about the consequences of not eating healthy foods (perceived severity) to increase the likelihood and cues for action.^18^ The Social Cognitive Theory constructs and activities incorporated into the program included self‐efficacy, observational learning, behavioural capability, cognitive restructuring, social support, and goal setting^19^ (refer to Table 1).

**TABLE 1:** Behaviour change technique mapping to Food Sensations® for Adults program curriculum.

Overall Food Sensations® for Adults program curriculum learning outcomes
Increased number of participants who demonstrate: Understanding of the impact of food on personal wellbeing Positive attitudes towards healthy eating Food literacy knowledge, skills, and confidence Intentions to regularly select, prepare and eat nutritious foods including trying new foods

Module	Purpose	Learning objectives	Theory constructs and activities	Behaviour change techniques
Module 1—Healthy eating (60 min)	To provide participants an overview of healthy eating and demonstrate how these principles can be incorporated into everyday life using the recommendations from the national dietary guidelines	1. Categorise foods into the five core and discretionary food groups as outlined in the dietary guidelines 2. Understand the links between eating a variety of foods and nutrients for the prevention of chronic disease and the maintenance of good health 3. Utilise the dietary guidelines to determine the recommended number of serves, for their age and gender, of each food group 4. Set goals to motivate behaviour change for healthier eating	Cues for action (perceived benefits and health motivation)^39^ Perceived threat (perceived severity and susceptibility)^39^ Self‐efficacy and observational learning^40^ Behavioural capability^40^ Cognitive restructuring,^19^ for example, providing accurate information about nutrition recommendations and emphasising that healthy food is delicious and can fit with what people are used to eating Social support^40^ Goal setting,^40^ for example, participants to select and review goals related to lesson content	1.1 Goal setting (behaviour) 1.4 Action planning 1.9 Commitment 4.1 Instructions on how to perform the behaviour 5.1 Information about health consequences 5.3 Information about social and environmental consequences 6.1 Demonstration of the behaviour 7.1 Prompts/cues 9.1 Credible source
Module 2—Label reading and food selection (60 min)	To provide participants with the necessary knowledge to read food labels to help support the selection and consumption of nutritious foods	1. Demonstrate the skills required to read and interpret a food label to compare products based on health and price 2. Determine if a food product is a best, ok or poor choice for a specific nutrient (e.g., fat, sugar, salt, fibre) 3. Understand the links between eating a variety of foods and nutrients for the prevention of chronic disease and the maintenance of good health 4. Review goals to motivate the trial of healthier eating	Cues for action (perceived benefits and health motivation)^39^ Self‐efficacy and observational learning,^40^ for example, opportunity to practice cooking Behavioural capability^40^ Social support,^40^ for example, education in community setting Goal setting^40^	1.1 Goal setting (behaviour) 1.2 Problem solving 3.1 Social support (unspecified) 4.1 Instructions on how to perform the behaviour 5.1 Information about health consequences 6.1 Demonstration of the behaviour 8.1 Behavioural practice/rehearsal 9.1 Credible source 10.4 Social reward 12.5 Adding objects to the environment
Module 3—Budgeting and meal planning (60 min)	To enable participants to effectively plan meals, shop and cook to promote the trial of healthy and inexpensive eating	1. Use money saving strategies for food shopping 2. Develop a meal plan to effectively plan and manage a household menu and budget 3. Substitute ingredients to assist with meal planning and food budgeting 4. Understand the links between eating a variety of foods and nutrients for the prevention of chronic disease and the maintenance of good health 5. Review goals to motivate the trial of healthier eating	Cues for action (perceived benefits and health motivation)^39^ Perceived threat (perceived severity and susceptibility)^39^ Self‐efficacy and observational learning^40^ Behavioural capability,^40^ for example, skills to plan, select, prepare, and eat healthier meals Social support^40^ Goal setting^40^	1.1 Goal setting (behaviour) 4.1 Instructions on how to perform the behaviour. 5.1 Information about health consequences 5.3 Information about social and environmental consequences 6.1 Demonstration of the behaviour 6.2 Social comparison 7.1 Prompts/cues 8.1 Behavioural practice/rehearsal 9.1 Credible source 10.7 Self‐incentive 13.1 Identification of self as role model
Module 4—Food safety, preparation, and cooking (90 min weekly)	To enable participants to have a hands‐on experience develop or practice basic cooking and food safety skills as a means of motivating the adoption of a healthier lifestyle	1. Identify basic food safety hygiene, preparation and storage practices 2. Prepare at least one new recipe (meal or snack) 3. Learn and practice a wide range of basic. cooking skills and techniques 4. Understand the benefits of cooking and sharing food	Cues for action (perceived benefits and health motivation)^39^ Perceived threat (perceived severity and susceptibility)^39^ Self‐efficacy and observational learning,^40^ for example, opportunity to practice cooking, food preparation skills, and dishing up appropriate serving sizes and types of food Behavioural capability^40^ Social support^40^ Goal setting^40^	3.2 Social support (practical) 4.1 Instructions on how to perform the behaviour 5.1 Information about health consequences 6.1 Demonstration of the behaviour 8.1 Behavioural practice/rehearsal 8.3 Habit formation 9.1 Credible source 10.4 Social reward
Optional modules—Eating on the move, eating snacks, eating out, eating junk food, eating mindfully	To provide participants with the opportunity to evaluate their personal food choices and behaviours and identify strategies to maintain or improve health and wellbeing	1. Identify components of a healthy lunch, snack, commercial meal 2. Practice food label reading skills to choose healthier foods 3. Review goals to motivate the continued trial of healthier eating	Cues for action (perceived benefits and health motivation)^39^ Perceived threat (perceived severity and susceptibility)^39^ Self‐efficacy and observational learning^40^ Behavioural capability^40^ Social support^40^ Goal setting^40^	1.1 Goal setting (behavioural) 1.2 Problem solving 3.1 Social support (unspecified) 4.1 Instructions on how to perform the behaviour 5.1 Information about health consequences 5.3 Information about social and environmental consequences 6.1 Demonstration of the behaviour 7.1 Prompts/cues 8.2 Behaviour substitution 9.1 Credible source 12.1 Restructuring the physical environment 12.2 Restructuring the social environment

In 2015, the Food Sensations® for Adults program was updated, and a logic model was developed to correspond with the Best Practice Criteria for Food Literacy Programs^20^ and aligned to an Australian food literacy model covering skills in planning, management, selection, preparation, and eating,^7^ strengthening the focus on behaviour change with goal setting embedded in program delivery. Delivery of Food Sensations® for Adults included four core modules on healthy eating, label reading, budgeting, meal planning, and food safety once a week over 3 weeks, and offered an optional module in week four, including eating healthy eating on the move, eating snacks, eating out, eating junk food, and eating mindfully to contextualise food literacy to specific groups (program structure is available as Figure S1). Education was delivered through interactive activities in the first hour. The participants then cooked and tasted pre‐selected recipes from the Foodbank WA recipe booklets over the last hour and a half of every week. Goal setting was a major component of the program. Facilitators would assist participants to write one short term goal based on the lesson content and a longer‐term goal at the end of the first session. The facilitators would revisit participant goals at the beginning of remaining sessions and guide discussion about progress, enablers, and barriers. Participants were encouraged to refine and add to their goals at end of sessions two through four.

This cross‐sectional study consisted of two phases. The first phase used consensus methods to identify and map behaviour change techniques, and the second stage applied content analysis of process evaluation data to align the targeted behaviours and behaviour change techniques collected from participants between 2016 and June 2021.

A retrospective behaviour change technique review of the lesson content was conducted in 2021 by a team of seven public health nutritionists and dietitians, primarily responsible for program implementation and session delivery. All team members completed the Behaviour Change Technique Taxonomy v1 online training prior to review.^26^ Four core modules within the program curriculum were included in this review. The common objectives of the optional modules offered in week four of the program were mapped. The program facilitators operated according to a detailed facilitator manual to ensure fidelity of implementation. Team members reviewed the lesson plan component for each core and optional module independently and recorded the precise location and code of the specific behaviour change techniques identified (refer to table: https://digitalwellbeing.org/wp‐content/uploads/2016/11/BCTTv1_PDF_version.pdf). Independent coding was followed by team discussion on four occasions to corroborate the findings. Where at least five out of seven team members reached an agreement on behaviour change technique presence, the behaviour change technique location and number were recorded in a master matrix. The behaviour change technique matrix generated by the program facilitators was then cross‐checked by an independent program evaluator who had completed the 4‐week online University College London Behaviour Change Interventions short course.^26^ Each behaviour change technique assigned by the facilitation team was interrogated for correct classification to ensure that there was no overlap or oversubscription for any activity. Where there were disagreements regarding a behaviour change technique identification by the external evaluator, a core team of three original team members reviewed and made the final decision. Behaviour change techniques are presented in terms of numbers and short titles.

The Food Sensations® for Adults program was evaluated using pre‐ and post‐program participant questionnaires containing process and impact evaluation questions. The questions reflected the funder's commissioning service level outcomes and were informed by the evaluation plan (including a program logic model) approved by the funder. The process evaluation collected in the post‐program questionnaire were four open‐ended questions including the goals set by the participants at the start of the program (up to three items could be listed) and the changes they made (up to three items could be listed). Participants were also asked what they liked about the program and areas for improvement (they could list up to three topics/areas for both like and improvements). Both pre‐ and post‐questionnaires included a 13‐item food literacy behaviour checklist and two short questions on dietary behaviours (vegetables and fruit) to measure changes in the targeted behaviours. The development and validation of the food literacy behaviours checklist have been published elsewhere^27^, ^28^ and the results of the impact and follow‐up evaluations have been published.^23^, ^24^, ^25^ The questionnaires were trialled with the early program participants in 2016 for inclusion in the final questionnaire and participants self‐reported and were encouraged to complete all questions. The pre‐program questionnaire included eight sociodemographic variables: sex, age, highest educational level, household composition, postcode, birth in Australia, and identification as an Aboriginal and/or Torres Strait Islander. Postcode was converted to Socioeconomic Index for Areas decile using Australian Bureau of Statistics data and group into low (decile 1–4), middle (decile 5–7) and high socioeconomic areas (decile 8–10) as a proxy for income.^29^

Ethics approval was obtained from a Human Research Ethics Committee. The SQUIRE 2.0 (Revised Standards for Quality Improvement Reporting Excellence) Checklist for quality improvement research was also completed. A verbal explanation of the research purpose was provided to all participants at the start of the program (Week 1) as well as a written research information sheet. Consent was obtained if the participants completed the pre‐program questionnaire. At the end of the program (Week 4), participants were invited to complete the post‐program questionnaire. The questionnaires took approximately 5 min to complete. Between April 2016 and June 2021, there were 6113 participants in total with 4697 providing some evaluation response (76.8%). Both pre‐ and post‐program questionnaires were completed by 3032 participants, with 1111 participants only completing pre‐questionnaires and 564 participants only completing post‐questionnaires. participants, respectively. Ethics approval was obtained from the Human Ethics Committee of Curtin University (RDHS‐52‐16).

Content analysis was applied to the qualitative responses collected to develop objective inferences about responses to the process evaluation questions.^30^ Each response was coded, and similar comments were then joined into categories by a research assistant who maintained a coding framework. The coding framework contained an overall code number and title for a category with all similar participant responses recorded to enable consistent coding for the SPSS analysis file. The external evaluator reviewed the codes and subsequent categories and clarified any ambiguity. Each completed post‐questionnaire was manually coded for entry into SPSS using the coding framework. If the research assistant considered a new category had emerged to be added to the coding framework this was confirmed with the external evaluator. In response to goals set at the start of the program, 103 responses were coded into 19 categories presenting specific or generalised comments such as ‘eat more fruits and vegetables’. The same goal responses were then used to code the self‐reported changes made by participants at the end of the program. Participants could provide up to three responses for what they liked about the program and suggestions for improvements. In 103 responses from comments on what they liked most about the program, 15 response categories resulted from coding, such as ‘recipe books’ and 53 were coded for program improvement suggestions, resulting in 13 categories. The goals and change categories were aligned with the dietary and food literacy components as targeted behaviours. The self‐reported data collected regarding program responses were aligned with the behaviour change techniques identified in the mapping by the behaviour change technique mapping team and an external evaluator. Statistical analysis was applied to those participants registering setting goals and making dietary changes versus those who did not set goals or make changes. Food Sensations® for Adults' three food literacy behaviour change factor scores, Plan & Manage, Selection and Preparation, and the two self‐reported dietary behaviours, serves of fruit and serves of vegetables, were treated as continuous variables and compared using ANCOVA. Statistical significance was set at *p* < 0.05. The participant's demographic characteristics: Sex and age group, Socio‐Economic Indexes for Areas (SEIFA index), household structure, education level, employment status, Australian‐born, identified as Aboriginal or Torres Strait Islander categories, were compared using the chi‐square test between those who completed both pre‐ and post‐questionnaires versus those only completing pre‐questionnaires. If participants only completed post‐questionnaires, then some process evaluation data were available for analysis but no demographic characteristics were available.

## 3 RESULTS

Mapping produced 22 different behaviour change techniques across the core education modules, optional education modules, and food safety, preparation, and cooking modules (Table 1). Nine behaviour change techniques were used weekly, four behaviour change techniques appeared more than once over 4 weeks and nine additional behaviour change techniques were mapped once over the course of the education (Table 2).

**TABLE 2:** Frequency of use of behaviour change techniques.

Behaviour change techniques used weekly	Behaviour change techniques used more than once over 4 weeks	Behaviour change techniques used only once
1.1 Goal setting (behaviour) 3.2 Social support (practical) 4.1 Instructions on how to perform the behaviour 5.1 Information about health consequences 6.1 Demonstration of the behaviour 8.1 Behavioural practice/rehearsal 8.3 Habit formation 9.1 Credible source 10.4 Social reward	1.2 Problem solving 3.1 Social support (unspecified) 5.3 Information about social and environmental consequences 7.1 Prompts/cues	1.4 Action planning 1.9 Commitment 6.2 Social comparison 8.2 Behaviour substitution 10.7 Self‐incentive 12.1 Restructuring the physical environment 12.2 Restructuring the social environment 12.5 Adding objects to the environment 13.1 Identification of self as a role model

The demographic characteristics are presented as those who completed pre‐ and post‐questionnaires compared with participants who only completed pre‐questionnaires. Most participants were female (Table 3) and aged between 26 and 35 years of age and from low to middle SEIFA index areas. The most common household structures were living with partners and/or children. Most participants had a certificate/diploma, were either part‐time or casual, or were not currently working. Nearly two‐thirds of the participants were born in Australia. There were statistically significant differences in characteristics between those who completed pre‐ and post‐questionnaires versus pre‐questionnaire only for age, household structure, education level, employment status and identifying as Aboriginal or Torres Strait Islander. The characteristics that were not statistically significant were sex, SEIFA index and being born in Australia.

**TABLE 3:** Participants demographic characteristics (2016 to June 2021).

Characteristic	Responses	Pre‐ and post‐questionnaires	Pre‐questionnaire only
Sex^a^	Female	2197 (72.8)	766 (70.1)
	Male	817 (27.1)	325 (29.7)
	Other	5 (0.2)	2 (0.3)
	Total sample	*N* = 3019	*N* = 1093
Age^b^	18–25 years	314 (10.6)	179 (16.8)
	26–35 years	607 (20.4)	272 (25.5)
	36–45 years	591 (19.9)	214 (20.1)
	46–55 years	425 (14.3)	136 (12.8)
	56–65 years	400 (13.4)	128 (12.0)
	66 and over	639 (21.5)	136 (12.8)
	Total sample	*N* = 2976	*N* = 1065
Socioeconomic Index for areas^c^	Low deciles	1160 (42.8)	402 (43.3)
	Middle deciles	806 (29.7)	300 (32.3)
	High deciles	746 (27.5)	227 (24.4)
	Total sample	*N* = 2712	*N* = 929
Household structure^b^	Live with partner and children	866 (29.1)	307 (28.7)
	Live with partner	669 (22.5)	184 (17.2)
	Live alone	597 (20.1)	189 (17.7)
	Single parent with child/children	257 (8.6)	111 (10.4)
	Shared accommodation	237 (8.0)	133 (12.5)
	Live with family/extended family	273 (9.2)	114 (10.7)
	Supported accommodation	78 (2.6)	30 (2.8)
	Total sample	*N* = 2977	*N* = 1068
Education level^b^	Certificate/diploma	890 (30.2)	284 (26.8)
	Some secondary/primary	626 (21.2)	292 (27.6)
	Bachelor or higher	678 (23.0)	206 (19.5)
	Finished high school	594 (20.2)	99 (17.6)
	Trade/apprenticeship	159 (5.4)	61 (5.8)
	Total sample	*N* = 2949	*N* = 1095
Employment status^b^	Part‐time or casual	1055 (35.7)	389 (36.7)
	Not currently working	459 (15.5)	171 (16.1)
	Unemployed	339 (11.5)	163 (15.4)
	Full‐time	340 (11.5)	143 (13.5)
	Unable to work/disability	764 (25.8)	153 (27.2)
	Total sample	*N* = 2957	*N* = 1061
Born in Australia^d^		1795 (61.2)	639 (63.0)
	Total sample	*N* = 2870	*N* = 557
Identify as Aboriginal or Torres Strait Islander^b^		217 (7.5%)	133 (13.4%)
	Total sample	*N* = 2879	*N* = 994

^a^ Sex *p* = 0.236.

^b^ *p* <0.001.

^c^ SEIFA *p* = 0.136.

^d^ Born in Australia *p* = 0.294.

Table 4 shows the pre‐ and post‐program changes reported by the participants. Participants recorded 2567 pre‐program goals on the post‐questionnaire, the most common goal being to eat healthier foods (36.4%), change eating habits (18.7%), eat more fruits and vegetables (71.1%), and cook healthier meals (12.1%). At the end of the program, participants listed 2506 changes, and the major self‐reported changes made were eating more fruits and vegetables (23.6%), eating healthier foods (21.3%), changing eating habits (specific) (16.2%), and cooking healthier meals (8.0%) of the targeted behaviours.

**TABLE 4:** Goals and changes made due to program (2016 to June 2021).

Target behaviour	Category	Pre‐program goals^a^	Post‐program changes made^a^
		*n* (%)	*n* (%)
		*n* = 2567	*n* = 2506
Dietary intake	Eat healthier foods	680 (26.4)	388 (15.5)
	Change eating habits	349 (13.6)	295 (11.8)
	Eat more fruit and vegetables	319 (12.4)	430 (17.1)
	Reduce portion sizes	84 (3.3)	109 (3.3)
	Mindful eating	71 (2.7)	52 (2.1)
	Subtotal	1503 (58.6)	1274 (50.8)
Food literacy—Food preparation and cooking domain	Cook healthier meals	226 (8.8)	146 (5.8)
	Get/cook new recipes	218 (8.5)	140 (5.6)
	Learn more about cooking, improve cooking confidence	132 (5.1)	91 (3.6)
	Subtotal	576 (22.4)	377 (15.0)
Food literacy—Planning and managing, selection	Read food labels	131 (5.1)	254 (10.1)
	Practice meal planning	124 (4.8)	121 (4.8)
	Budget and save money	66 (2.6)	35 (1.4)
	Alter food shopping/selection	35 (1.4)	103 (4.1)
	Healthy lunchboxes and snacks	28 (1.1)	11 (0.4)
	Subtotal	384 (15.0)	524 (20.9)
Other	Improve health (specific), that is, lose weight, exercise	173 (6.7)	82 (4.5)
	Other (general learning, food safety, reduce waste, social aspects, assist family member, or client)	80 (3.1)	131 (7.2)
	Subtotal	253 (9.8)	213 (11.7)

^a^ Participants could write up to three goals or changes.

While dietary behaviours comprised just over half of the goals at the start of the program, by the end of the program, there was an equal split between the dietary and food literacy behaviours listed by the participants.

Seven percent of participants did not have any recorded goals or changes. For the remainder of the sample, Table 5 presents participants who were more likely to set goals and make changes. There was a clear pattern that females were statistically more likely to set goals and record changes (43.6% difference) (*p* < 0.001). Similarly, older age groups compared to younger (63.8%) (*p* < 0.001), living in a higher SEIFA area (62.8%) (*p* = 0.049), living with children compared to other household structures (60.8%) (*p* = 0.014), having a bachelor's degree compared to other education levels (*p* < 0.001), being born in Australia, and not identified as Aboriginal and/or Torres Strait Islander (*p* < 0.001). Goal setting and making changes were statistically significant for higher food literacy factor scores, Plan & Manage (*p* < 0.001), selection (*p* < 0.001), and preparation (*p* < 0.0001). Setting goals also produced statistical significance for the serves of fruit (*p* = 0.004) but not for vegetable serves (*p* = 0.192).

**TABLE 5:** Effect of demographic variables and food literacy scores and dietary behaviour change serves because of goal setting and making changes groups.

Variables		Goals not set—Changes not made^a^	Goals set—Changes made	Difference (95% CI)	*p*‐value
Demographic^b^					
Sex	Male	288 (35.8%, 28.2%)	734 (26.4%, 71.8%)	43.6% (40.6%–46.6%)	**<0.001**
	Female	516 (64.2%, 20.1%)	2048 (73.6%, 79.9%)	59.8% (57.9%–61.7%)	
Age, years	18–25	108 (14.8%, 29.0%)	264 (10.3%, 71.0%)	42.0% (37.0%–47.0%)	**<0.001**
	26–35	182 (24.9%, 25.9%)	521 (20.3%, 74.1%)	48.2% (44.5%–51.9%)	
	36–45	139 (19.0%, 21.0%)	524 (20.4%, 79.0%)	58.0% (54.2%–61.8%)	
	46–55	100 (13.7%, 21.6%)	363 (14.1%, 78.4%)	56.8% (52.3%–61.3%)	
	56–65	80 (11.0%, 18.5%)	353 (13.7%, 81.5%)	63.0% (58.5%–67.5%)	
	66+	121 (16.6%, 18.1%)	547 (21.3%, 81.9%)	63.8% (60.2%–67.4%)	
SEIFA index	Low SEIFA 1–4	248 (40.9%, 19.5%)	1026 (43.1%, 80.5%)	61.0% (58.3%–63.7%)	**0.049**
	Middle SEIFA 5–7	206 (34.0%, 23.0%)	689 (29.0%, 77.0%)	54.0% (50.7%–57.3%)	
	High SEIFA 8–10	152 (25.1%, 18.6%)	664 (27.9%, 81.4%)	62.8% (59.5%–66.1%)	
Household structure	Live alone	150 (20.4%, 22.8%)	509 (19.8%, 77.2%)	54.4% (50.6%–58.2%)	**0.014**
	Live with others (adults)	344 (46.7%, 24.4%)	1068 (41.6%, 75.6%)	51.2% (48.6%–53.8%)	
	Live with children	242 (32.9%, 19.6%)	990 (38.6%, 80.4%)	60.8% (58.1%–63.5%)	
Education level	Some secondary	222 (30.7%, 30.7%)	500 (19.6%, 69.3%)	38.6% (35.0%–42.2%)	**<0.001**
	Finished high school	128 (17.7%, 19.8%)	519 (20.4%, 80.2%)	60.4% (56.6%–64.2%)	
	Trade/apprenticeship	49 (6.8%, 26.5%)	136 (5.3%, 73.5%)	47.0% (39.8%–54.2%)	
	Certificate or diploma	199 (27.5%, 20.2%)	785 (30.8%, 79.8%)	59.6% (56.5%–62.7%)	
	Bachelor degree or higher	119 (16.5%, 16.5%)	604 (23.7%, 83.5%)	67.0% (63.6%–70.4%)	
	Other/never	6 (0.8%, 54.5%)	5 (0.2%, 45.5%)	−9.0% (−7.9% to 25.9%)	
Employment status	Full‐time	99 (13.7%, 25.0%)	297 (11.6%, 75.0%)	50.0% (45.1%–54.9%)	**<0.001**
	Part‐time or casual	176 (24.4%, 22.3%)	614 (24.0%, 77.7%)	55.4% (51.9%–58.9%)	
	Unemployed	165 (22.9%, 26.4%)	459 (17.9%, 73.6%)	47.2% (43.3%–51.1%)	
	Unable to work/disability	75 (10.4%, 26.7%)	206 (8.1%, 73.3%)	46.6% (40.8%–52.4%)	
	Not currently working	207 (28.7%, 17.4%)	982 (38.4%, 82.6%)	65.2% (62.5%–67.9%)	
Born in Australia	Yes	485 (65.5%, 24.0%)	1535 (60.7%, 76.0%)	52.0% (49.8%–54.2%)	**0.019**
	No	256 (34.5%, 20.5%)	995 (39.3%, 79.5%)	59.0% (56.3%–61.7%)	
Identify as Aboriginal and/or Torres Strait Islander	Yes	105 (14.6%, 37.1%)	178 (7.2%, 62.9%)	25.8% (20.7%–30.9%)	**<0.001**
	No	614 (85.4%, 21.0%)	2310 (92.8%, 79.0%)	58.0% (56.2%–59.8%)	

Behaviours^c^		Mean ± SD^c^	Mean ± SD	Difference (95% CI)	*p*‐value
Plan manage factor score	Pre	9.9 ± 2.4	9.6 ± 2.3	0.52 (0.27–0.77)	**<0.001**
	Post	10.3 ± 2.2	10.6 ± 2.0		
Selection factor score	Pre	6.4 ± 2.0	5.4 ± 2.4	0.52 (0.30–0.74)	**<0.001**
	Post	5.8 ± 2.5	6.2 ± 2.3		
Preparation factor score	Pre	13.0 ± 3.6	11.1 ± 4.5	0.83 (0.46–1.20)	**<0.001**
	Post	11.8 ± 4.7	12.5 ± 4.5		
Serves of fruit	Pre	1.6 ± 1.1	1.6 ± 1.1	0.20 (0.06–0.34)	**0.004**
	Post	1.7 ± 1.0	1.8 ± 1.0		
Serves of vegetables	Pre	2.4 ± 1.3	2.4 ± 1.3	0.12 (−0.06 to 0.29)	0.192
	Post	2.9 ± 1.4	2.9 ± 1.2		

*Note:* The *p* values are bolded when statistically significant.

Abbreviation: SEIFA Index, Socio‐Economic Indexes for Areas.

^a^ Cells for represent count (% within column, % within row) difference (95% CI) represent difference of percentages within row and associated 95% confidence interval.

^b^ Demographic categorical variables analysed using the chi‐square test.

^c^ Continuous variables analysed with ANCOVA, Cells represent mean ± standard deviation; difference (95% CI) represents difference between Post means after adjusting for Pre as covariate, and associated 95% confidence interval.

Participants' responses to what they liked about the program (*n* = 3819) resulted in 15 categories that were linked to recurring behaviour change techniques and certain behaviour change technique combinations (Table S1). There was strong support for the cooking and eating activities in the program (30% of responses), which contributed to 1.2 Problem solving, 3.1 Social support, 6.1 Demonstration of the behaviour and 8.1 Behavioural practice/rehearsal. Education materials used, such as recipes and recipe books contributed to 12.5 Adding objects to the environment in addition to the group learning environment (3.2 Social support), the general learning experience, and the facilitators as the 9.1 credible source. Participants reported enjoying practical learning skills and increased confidence, which can be attributed to the activity structure where information is provided (5.1), followed by instruction and demonstration of the activity (4.1, 6.1, respectively). The participants then completed the activity (8.1) and were given resources to assist (12.5).

Participants were also asked to suggest improvements to the program. There were 2379 suggestions. Of these, 62.7% suggested no change or that the program was positive. The other suggestion was to have a longer program (9.6%) to include more recipe variety, such as single person recipes (6.6%) and more program availability (same or different location [5.5%]).

## 4 DISCUSSION

This is the first Australian study to elucidate the contribution of behaviour change techniques, specifically goal setting, to food literacy and dietary behaviour change in an established effective program.^25^ There was consistent use of nine behaviour change techniques in the program, supported by 13 other behaviour change techniques. The process evaluation demonstrated that the types of behaviours chosen by participants from behaviour change technique 1.1 goal setting, self‐reported change responses, food literacy, and dietary behaviour change were approximately equal. Goal setting had a statistically significant association with higher food literacy frequency of behaviours and fruit servings. There was evidence of single and combinations of behaviour change techniques reflected in open‐ended comments on what the participants liked about the program. These findings indicate that the Food Sensations® for Adults program successfully applied multiple behaviour change techniques adopted by participants to support food literacy and dietary behaviour changes.

The number of behaviour change techniques identified in published nutrition education programs varies. There is evidence that behaviour change techniques identified in general brief nutrition education programs range from 0 to 14, with a median of three.^31^ The 2018 review of cooking skill programs identified between one and 11 behaviour change techniques, although none explicitly referred to techniques as behaviour change techniques.^6^ Specifically, for food literacy programs, one effective parent and child vegetable cooking program used nine behaviour change techniques^17^ and a protocol paper for an 11 week digitally delivered food literacy program indicated the use of one to four behaviour change techniques per week, with a total of 13 behaviour change techniques to be used.^18^ Food Sensations® for Adults has more behaviour change techniques than found previously by other limited reviews,^6^, ^8^, ^9^ and this is partly because the program involves nutrition education sessions in addition to food preparation and cooking sessions. The dual mode of delivery enables facilitators to focus on different behaviour change techniques, go beyond increasing knowledge (such as 5.1 Information on health consequences) by providing an opportunity to discuss, share, and practice skills and behaviours, and increase personal relevance to support techniques (like 1.1 Goal setting). While goal setting and making changes were statistically significant for food literacy behaviours and serves of fruit, it is likely that other behaviour change techniques were influential in improving the serves of vegetables, as demonstrated by a previous evaluation.^25^ We have shown that goal setting on its own does not necessarily work for all participants, including men, those from younger age groups, less educated, lower socioeconomic status, and different cultural groups, including Indigenous people, who are less likely to use goal setting. These results support the conclusion that a combination of behaviour change techniques is required to support the diversity of participants in changing food literacy and dietary behaviours in these types of programs.

Hollywood, Surgenor^6^ found that the four most frequently occurring behaviour change techniques evident in effective programs were 5.1: Provide information on consequences of behaviour; 4.1 Provide instruction on how to perform the behaviour, 6.1 Demonstrating behaviours and 8.1 Behavioural Practice/Rehearsal Hollywood, Surgenor.^6^ These behaviour change techniques also appear in the Food Sensations® for Adults program, but other programs vary in their use. Common to all food literacy program evidence is 1.1 Goal setting and 4.1 Instructions on how to perform the behaviours and 5.1 Information about health consequences. Importantly, the review found that interventions that used all four behaviour change techniques maintained behavioural changes in food literacy elements and diet beyond 3 months.^3^ Food Sensations® for Adults provides participants with information and skills on how to enact food literacy behaviours, such as planning meals, writing a shopping list, interpreting nutrient content on food labels, and focusing on budget‐friendly meals and snack ideas. The program has shown that behaviours are maintained or improved at 3‐month follow‐up.^24^

Despite the lack of robust evidence for food literacy program effectiveness, systematic review findings suggest that practical interventions with hands‐on cooking (most frequently applying behaviour change techniques 8.1 Behaviour practice/rehearsal, 4.1 Instruction on how to perform the behaviour and 12.5 Adding objects to the environment) appear to be associated with long‐term success.^6^, ^32^ Not all published program evaluations indicate a theory selection that informs the application of behaviour change techniques, and there may be a tendency to revert to common theories. Maugeri, Brimblecombe^15^ used realist synthesis to find the main mechanisms often identified in what was called nutrition education and cooking interventions, which were related to the prolific use of Social Cognitive Theory, for example, increased self‐efficacy from hands‐on practice, and therefore, may be overemphasising constructs from this theory.^15^ Further work/research with behaviour change techniques applications will assist in identifying theories that are better suited to food literacy programs.

While the Food Sensations® for Adults program is effective, based on facilitator feedback and program findings, there are additional behaviour change techniques that could further support behavioural changes in food literacy programs, and participants who required more support. Increasing opportunities for building motivation for change as people are exposed to building capability in their knowledge, skills, and physical opportunities for cooking and eating together in a social environment. For example, 1.2 Problem solving could be strengthened by consistently adding 1.4 Action planning to lesson plans to support people in identifying barriers such as time, budget, and household preferences. This would enable participants to tailor their solutions according to their personal circumstances. Opportunities exist to add rewards for behavioural change and further develop the social support aspects of cooking and eating in a group setting. Of the limited feedback on improvements to the Food Sensations® for Adults program, the proportion that wanted longer programs or more advanced programs is the recognition that behaviour change takes time and people will be at different stages in their change process, requiring different supports.

The challenges in identifying behaviour change techniques are that the effect of a single behaviour change technique may be very small and that many behaviour change techniques combine in program delivery and have the potential to amplify one another.^33^ A greater number of behaviour change techniques may not automatically lead to greater behavioural change, but it may be more important to consider the utility of individual behaviour change techniques.^34^ Behaviour change techniques are the smallest irreducible components, the active ingredients that facilitate behaviour change. The research on how behaviour change techniques relate to theoretical contracts or mechanisms of actions is still evolving. Program developers have many different theories of behaviour change to choose from that have similar and overlapping constructs.^35^ For example, goal setting is linked with behavioural regulation and information about health consequences with the constructs perceived susceptibility and intentions. More work needs to be done on elucidating how behaviour change theories can be selected for nutrition program design. This would inform the selection of behaviour change techniques for use in program implementation to assist with improvement and replication of effective programs.^36^ The research defining behaviour change techniques has also seen the emergence of the COM_B model (capability, opportunity, motivation to perform behaviour) which attempts to draw a number of constructs together into one theory of behaviour change for multiple contexts.^37^ Future food literacy programs would benefit from considering the COM‐B model which encompasses the behaviour change techniques identified but potentially would identify with the potential mechanism to identify further techniques to support behaviour change.

We also showed that some participants may need more assistance with goal setting as a behaviour change technique. Behaviour change techniques are only as good as the quality of program delivery and consistency when multiple facilitators are involved because of implementation fidelity considerations, which are also rarely reported.^21^ There is the possibility that there are other impacts of people coming together in community groups to learn, cook, and eat together that are not captured in behaviour change techniques and process evaluations. For example, Australian Jamie's Ministry of Food 10‐week cooking program found that participants reported greater household involvement in cooking and mealtimes at home, resulting in increased social interactions after the program.^38^ These social constructs supporting behaviour changes might be captured by applying a broader model such as the COM‐B model to develop a theory of change.

The Food Sensations® for Adults program mapping of behaviour change techniques has provided a model for designing future programs using behavioural science to apply a systematic method in developing interventions. In the future, determining the behaviour change techniques linked to newer comprehensive behaviour change models such as the COM‐B model, representing Capability, Opportunity, and Motivation leading to behaviour change^11^ and using these components to perform a behavioural diagnosis to understand all the determinants of food literacy behaviours linked to dietary behaviours will assist in the evolution of appropriate strategies for interventions. There is value in mapping behaviour change techniques when designing a program, but also when a program is running as per the Food Sensations® for Adults program, as it was found that mapping assisted in revising the language in the lesson plans to reinforce existing behaviour change techniques.

We acknowledge several limitations of the behaviour change technique mapping and process evaluation. While the authors applied a rigorous method to behaviour change technique mapping and used several iterations and checks to identify behaviour change techniques, some might have been missed. The process evaluation data were self‐reported by participants who attended the final week of the program or completed an online survey sent after the program completion. Not all participants recorded setting goals and making changes, and it could have been the more motivated participants to complete this part of the post‐program questionnaire.

The present study clearly demonstrated positive behavioural changes with the repeated use of behaviour change techniques across a food literacy program. Knowing the behaviour change techniques associated with effective programs can facilitate replication of new interventions. There is an urgent need for more rigorous evaluation to test program effectiveness and advance the application of behaviour change techniques in education‐ and training‐focused interventions. This would augment investment in programs by developing the best combination of behaviour change technique applications to maximise the potential efficacy of interventions. Future research should consider how to combine evidence from different types of evaluations to judge the likely effect sizes of behaviour change technique combinations, specifically for food literacy and subsequent dietary behaviour change.

## AUTHOR CONTRIBUTIONS

All the authors agree with the manuscript and declare that the content has not been published elsewhere. LMB and AB were involved in the conceptualisation and design of the study. Manuscript draft preparation was conducted by all authors. Results were analysed by LMB and AB. All authors provided review and editing.

The authors of this paper acknowledge the Foodbank WA program facilitators who assisted with the questionnaire processes and administering the questionnaires, including Vanessa Bobongie, Frances Foulkes‐Taylor, Kim Dutkowski, Michelle McIntosh, Nicole Ingram, Catherine Dumont, Amber Rose, Xavier Wong, and Nerissa Le. Thank you to Satvinder S. Dhaliwal for statistical advice.

## FUNDING INFORMATION

This study was funded as part of the research and evaluation of the Food Sensations® for Adults program conducted by Foodbank WA and funded by the Western Australian Department of Health grant number C06458. Open access publishing facilitated by Edith Cowan University, as part of the Wiley ‐ Edith Cowan University agreement via the Council of Australian University Librarians.

## CONFLICT OF INTEREST STATEMENT

Andrea Begley is an Associate Editor for Nutrition & Dietetics. No conflict of interest declared for other authors.

## Supporting information


Figure S1.

Table S1.

## Data Availability

These data are owned by the WA Department of Health and are not publicly available.
