# Supplementary figures and images for: Analysing the behaviour change techniques in an effective food literacy program to inform future program design
Source: Nutr Diet. 2024 Oct 22;82(3):268–82. doi: 10.1111/1747-0080.12908 (PMC12168058; doi:10.1111/1747-0080.12908)

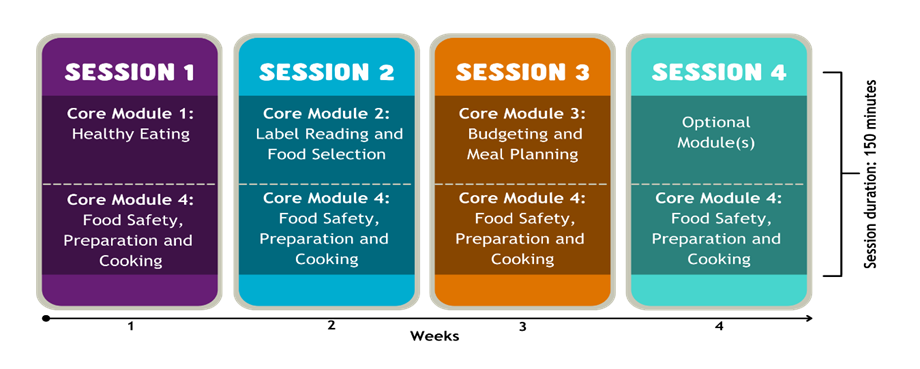


Supplementary Figure 1 FSA program curriculum

Supplement: Supplementary file 1 — Figure S1. [file NDI-82-268-s001.docx]
