# Supplementary material for: Analysing the behaviour change techniques in an effective food literacy program to inform future program design
Source: Nutr Diet. 2024 Oct 22;82(3):268–82. doi: 10.1111/1747-0080.12908 (PMC12168058; doi:10.1111/1747-0080.12908)
Supplement: Supplementary file 2 — Table S1. [file NDI-82-268-s002.docx]

**Supplementary Table 1 Recurring behaviour change techniques and behaviour change techniques combinations that align with participant open-ended comments about what they liked most about the program.**

| Behaviour change technique(s) | What participants liked most | Total delivery 2016-2021  (n=3819)  % |
| --- | --- | --- |
| 1.2 Problem solving | Participating in the cooking and eating activity | 1087 (28.5) |
|  | Learning new cooking skills / increasing cooking confidence | 245 (6.5) |
|  | Learning nutritional content of takeaway and sugary drinks | 40 (1.0) |
|  | Budgeting and meal planning topics | 168 (4.4) |
| 3.1 Social support (unspecified)  3.2 Social support (practical) | Participating in the cooking and eating activity | 1087 (28.5) |
|  | Learning new cooking skills / increasing cooking confidence | 245 (6.5) |
|  | Group interactions, sharing experiences and fun program | 595 (15.6) |
| 4.1 Instruction on how to perform the behaviour  5.1 Information about health consequences  6.1 Demonstration of the behaviour  8.1 Behavioural practice/rehearsal  12.5 Adding objects to the environment | Participating in the cooking and eating activity | 1087 (28.5) |
|  | Reading food labels | 310 (8.1) |
|  | Learning new cooking skills/increasing cooking confidence | 245 (6.5) |
|  | Budgeting and meal planning topics | 168 (4.4) |
|  | Learning nutritional content of takeaway and sugary drinks | 40 (1.0) |
|  | Program activities and structure (general) | 183 (4.8) |
|  | Motivation, renewed confidence | 93 (2.4) |
| 5.1 Information about health consequences | Learning about healthy eating nutrition | 531 (13.9) |
|  | Education, information, and learning (generally) | 529 (13.9) |
| 8.3 Habit formation | Participating in the cooking and eating activity | 1087 (28.5) |
|  | Reading food labels | 310 (8.1) |
|  | Learning new cooking skills/increasing cooking confidence | 245 (6.5) |
| 12.5 Adding objects to the environment | Recipe books | 687 (18.0) |
|  | Resources (specific and general) | 136 (3.6) |
| 9.1 Credible source | Great facilitators | 416 (10.9) |
| Too general to map to a behaviour change technique | Great program, useful, liked everything | 320 (8.4) |
|  | Other (variety of small responses) | 138 (3.6) |
